# Waardenburg syndrome type 2A in a large Iranian family with a novel *MITF* gene mutation

**DOI:** 10.1186/s12920-021-01074-y

**Published:** 2021-09-20

**Authors:** Safoura Zardadi, Sima Rayat, Maryam Hassani Doabsari, Mohammad Keramatipour, Saeid Morovvati

**Affiliations:** 1 Department of Biology, School of Basic Sciences, Science and Research Branch, Islamic Azad University, Tehran, Iran; 2 Tehran Medical Sciences, Islamic Azad University, Tehran, Iran; 3 Department of Medical Genetics, Faculty of Medicine, Tehran University of Medical Sciences, Tehran, Iran; 4 Department of Genetics, Faculty of Advanced Sciences and Technology, Tehran Medical Sciences, Islamic Azad University, Tehran, Iran

**Keywords:** Waardenburg syndrome, *MITF*, Hearing loss

## Abstract

**Background:**

The characteristics of Waardenburg syndrome (WS) as a scarce heritable disorder are sensorineural hearing loss and deficits of pigmentation in the skin, hair, and eye. Here, clinical features and detection of the mutation in the *MITF* gene of WS2 patients are reported in a sizable Iranian family.

**Methods:**

A man aged 28-years represented with symptoms of mild unilateral hearing loss (right ear), complete heterochromia iridis, premature graying prior to 30 years of age, and synophrys. In this research, there was a sizable family in Iran comprising three generations with seven WS patients and two healthy members. Whole exome sequencing was applied for proband for the identification of the candidate genetic mutations associated with the disease. The detected mutation in proband and investigated family members was validated by PCR-Sanger sequencing.

**Results:**

A novel heterozygous mutation, NM_198159.3:c.1026dup p.(Asn343Glufs*27), in exon 9 of the *MITF* gene co-segregated with WS2 in the affected family members. The variant was forecasted as a disease-causing variant by the Mutation Taster. According to the UniProt database, this variant has been located in basic helix-loop-helix (bHLH) domain of the protein with critical role in DNA binding.

**Conclusions:**

A frameshift was caused by a nucleotide insertion, c.1026dup, in exon 9 of the *MITF* gene. This mutation is able to induce an early termination, resulting in forming a truncated protein capable of affecting the normal function of the MITF protein. Helpful information is provided through an exactly described mutations involved in WS to clarify the molecular cause of clinical characteristics of WS and have a contribution to better genetic counseling of WS patients.

**Supplementary Information:**

The online version contains supplementary material available at 10.1186/s12920-021-01074-y.

## Background

The characteristics of Waardenburg syndrome (WS) as a scarce heritable disorder are sensorineural hearing loss and deficits of pigmentation in the skin, hair, and eye. The four types of WS (WS1-WS4) are based on genetic criteria and whether additional symptoms are present or absent. Although the features of type 1 (OMIM 193500) and type 2 (OMIM 193510) are highly similar clinically, type 1 is distinguished from type 2 by the presence of dystopia canthorum (an outward displacement of the inner canthi of the eyes). Besides clinical features of type 1, type 3 (OMIM 148820) comprises musculoskeletal abnormalities. The characteristics of type 4 (OMIM 277580) are similar to those of type 2 with the addition of Hirschsprung disease. Mutations in six genes, namely melanocyte inducing transcription factor (*MITF*), SRY-box transcription factor 10 (*SOX10*), snail family transcriptional repressor 2 (*SNAI2*), endothelin receptor type B (*EDNRB*), endothelin 3 (*EDN3*), and paired box 3 (*PAX3*), can cause WS. Mutations in *PAX3* gene cause types 1 and 3, type 2 is linked to defective *MITF* and *SNAI2* genes, and mutations in *EDN3* and *EDNRB* genes have associations with type 4 while some types 2 and 4 cases result from the mutations in the *SOX10* gene [1–6].

As a heterogeneous disorder, WS2 is grouped into five subtypes according to the genetic cause. Varying degrees of sensorineural hearing loss and pigmentary disturbances of skin, hair and eye characterize WS2A (OMIM 193510) as an autosomal dominant disorder. WS2A is caused by heterozygous mutations in the *MITF* gene on chromosome 3p14.1-p12.3 and such clinical features are caused by deficiency of normal melanocytes in the affected organs including the eye, skin, and cochlea [2, 7–10]. The microphthalmia (MiT) family of transcription factors consists of MITF, TFEB, TFEC, and TFE3, sharing a joint basic-helix-loop-helix-leucine zipper (bHLHZip) dimerization motif and a transactivation domain (TAD). A *MITF* gene alone produces nine differing MITF isoforms, all of which share exons 2–9. The expression of MITF occurs in melanocytes and comprises a basic domain for DNA binding and a HLHZip domain for homo- or heterodimerization with either TFEB, TFEC or TFE3, recognizing special DNA sequences of pigmentation genes, namely tyrosinase (TYR), tyrosinase-related protein 1 (TRP1), and DCT/TRP2 that are involved in melanogenesis and regulate the expression of these genes, hence having a major contribution to developing and differentiating melanocytes [7, 11–14].

Despite the identification of abundant mutations in the *MITF* gene linked to WS2A in various ethnical groups, there are scarce reports in the population of Iran [6, 15–19]. Here, clinical manifestations and mutation detection of the *MITF* gene are reported in a sizable family in Iran with Waardenburg syndrome type 2A.

## Methods

### Patients

**Fig. 1:**
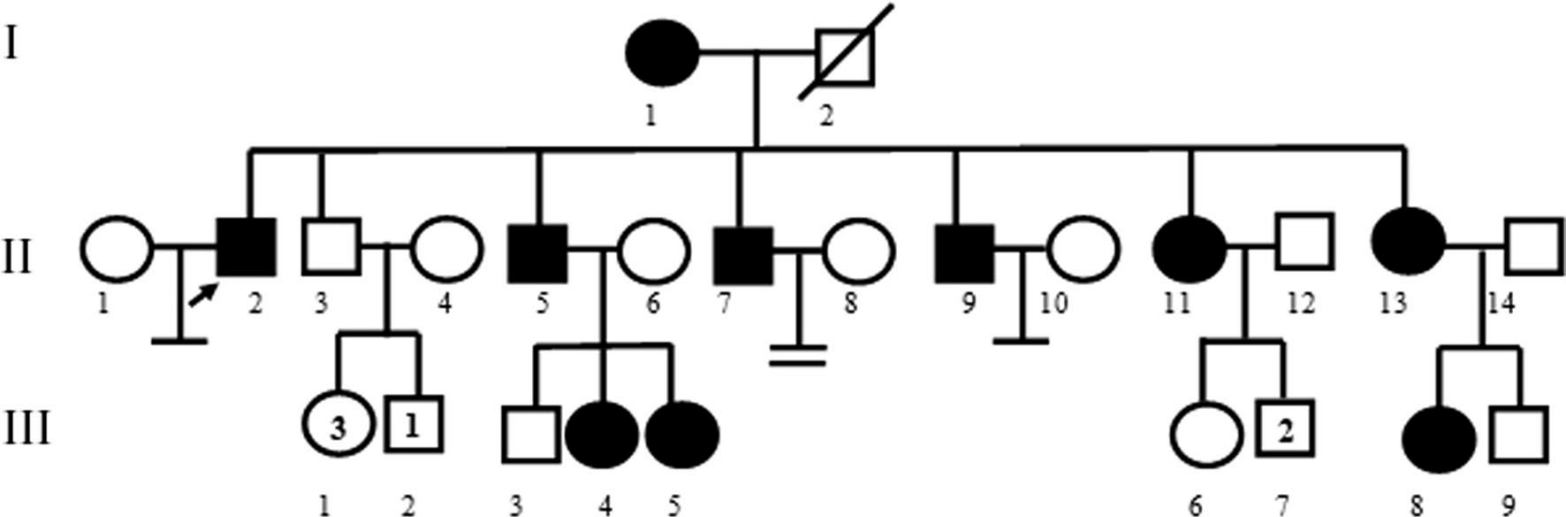
Pedigree of a family with Waardenburg syndrome type 2 with *MITF* mutation. The black arrow indicates the proband (II-2). Each generation is identified by a Roman numeral (I, II, III) and each individual within the same generation is identified by an Arabic numeral (1, 2, 3 and so on). The numbers inside symbols represent number of individuals. Squares represent males and circles represent females. The symbol with a diagonal line shows a deceased individual. Individuals’ symbols are colored black to indicate the presence of clinical characteristics of WS2, unfilled symbols represent unaffected family members

A 28-year-old symptomatic male referred to Rasad Pathobiology and Genetic Laboratory, Tehran, Iran for genetic counseling. Comprehensive clinical information was obtained and his affected family members thoroughly underwent clinical examinations. In this investigation, there was a sizable Iranian family comprising three generations with seven WS patients and two healthy members. Clinically, the signs were suggestive of diagnosing WS for the proband. Following genetic counseling, a familial pedigree was drawn (Fig. 1) and written informed consent was obtained from all the individuals included in this study.

**Fig. 2:**
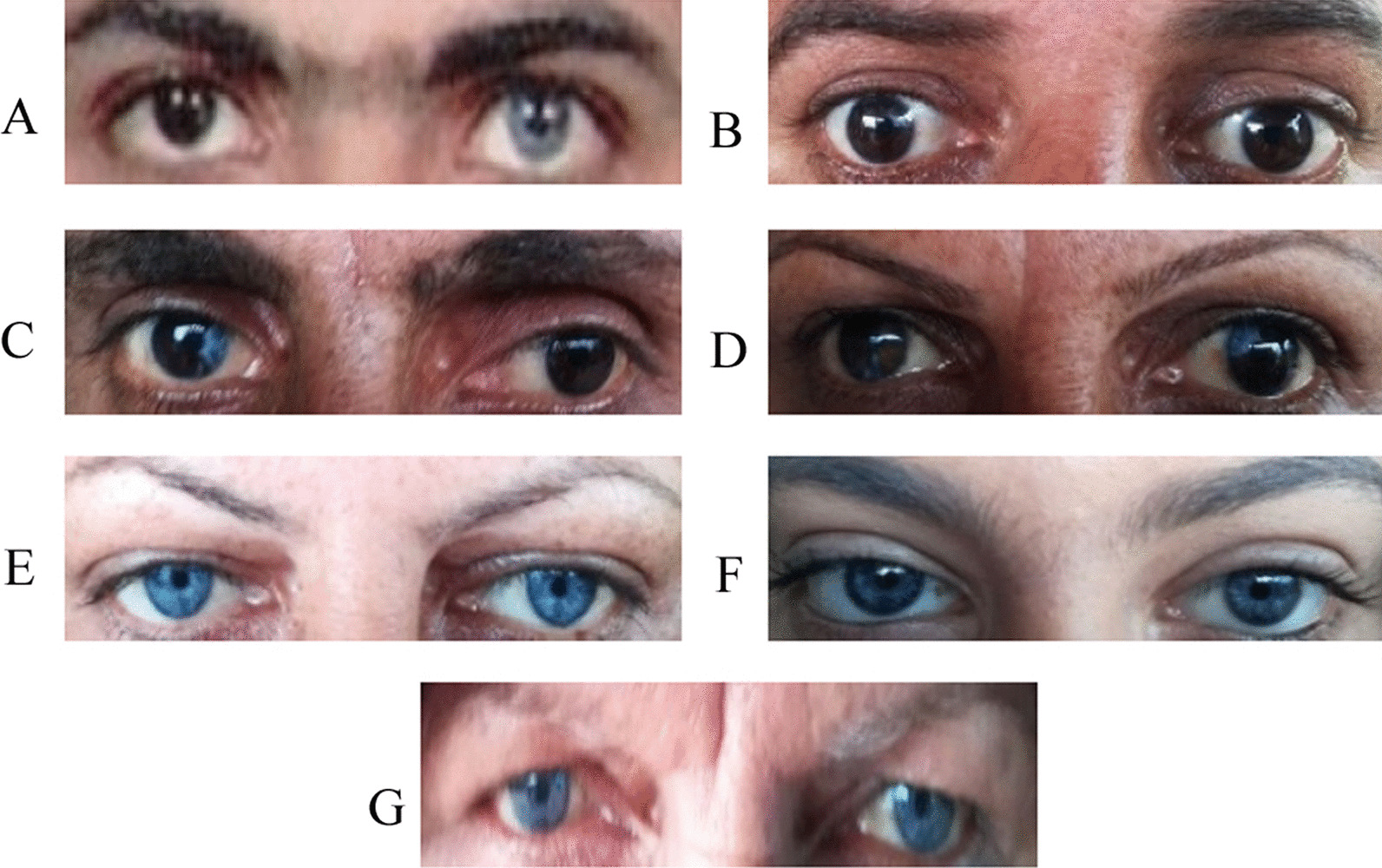
Iris color of Waardenburg syndrome type 2 patients. **A** II-2, complete heterochromia iridis. **B** II-7, a blue segment in his right iris. **C** II-9, blue segments in both irises. **D** II-11, brown and blue segments in her right iris and a blue segment in her left iris. **E** II-13, **F** III-8 and **G** I-1, brilliant blue irises

Mild unilateral hearing loss (right ear), complete heterochromia iridis, premature graying prior to age 30 and synophrys were observed in the proband (II-2). White frontal forelock and premature graying were found in his sibling (II-5). Mild unilateral hearing loss (right ear), a blue segment on his right iris, premature graying, and freckles on the face were present in one male sibling (II-7). Bilateral profound hearing loss, premature graying before age 30, synophrys, and plentiful facial brown freckles were visible in his brother (II-9) and his sister (II-11). The former manifested blue segments in his eyes but the latter represented brown and blue segments in her right iris and a blue segment in her left iris. There were similar phenotypes, namely mild unilateral hearing loss (right ear), brilliant blue eyes, synophrys, and facial freckles in two affected individuals (II-13 and III-8). Profound bilateral hearing loss, brilliant blue eyes, and ample abdominal brown freckles were observable in proband’s mother (I-1) (Fig. 2). Profound bilateral hearing loss, brilliant blue eyes and brown facial freckles were detected in two proband’s nieces (III-4 and III-5). One male sibling (II-3) was healthy. His parents were not consanguine.

### Mutation detection

The proband’s sample was subjected to whole exome sequencing (WES) to identify the causative gene or genes. Blood sample was obtained from the proband. Standard extraction procedures were used for genomic DNA extraction from whole blood. Library preparation was performed using Twist Core Exome kit (TWIST Bioscience, USA, https://www.twistbioscience.com/) through the manufacturer’s instruction. Sequencing of libraries was performed by high-throughput paired-end sequencing using NovaSeq sequencing platform (Illumina Inc., CA, USA). Interpretation of detected variants in terms of pathogenicity was done based on American College of Medical Genetics and Genomics (ACMG) guideline. The identified variant was evaluated for disease-causing nature using the Mutation Taster (http://www.mutationtaster.org) and UniProt database (www.uniprot.org).

The identified variant by WES was confirmed by PCR-sanger sequencing in the patient and then it was analyzed in several affected and healthy members of the family by PCR-sanger sequencing.

### PCR-Sanger sequencing

The mutation detected by WES within exon 9 of the *MITF* gene was investigated in patient and members of his family (I-1, II-2, II-3, II-7, II-9, II-11, II-13, III-7, and III-8) using PCR-Sanger sequencing technique with special primers. MITF-F: 5′-AGAAATGGAGTGCTTTGAATA TTG-3′ and MITF-R: 5′-CAAGAATGACTGTGGAAGTGC-3′. The PCR mixture was set totally in a 25-µl volume. All PCR amplifications were performed using 12.5 µl of PCR master mix (Yekta Tajhiz Azma, Iran), 8.5 µl of H_2_O, 1 µl of each primer, and 2 µl of extracted DNA. The reaction was begun initially with denaturation at 95 °C for 5 min, and then 35 cycles at 95 °C for 30 s, annealing at 59 for 45 s, and extension at 72 °C for 45 s, with finally an extension at 72 °C for 7 min on a thermal cycler (Veriti, Applied Biosystems, USA). To analyze the PCR products, they were run on 2% agarose gel, and then subjected to purification, followed by sequencing on an ABI 3500 Genetic analyzer.

## Results

### Molecular findings

By analyzing the sequencing data, a heterozygous variant, c.1026dup p.(Asn343Glufs*27), was detected in exon 9 of the *MITF* gene, having a perfect match with the patient's phenotype (Table 1). Mutation Taster predicted this variant as a disease causing variant. According to the UniProt database, the location of this variant is on basic helix-loop-helix (bHLH) domain of the protein. The bHLH motif is well-established functional domain with important role in DNA binding.

**Table 1:** Identified variant in this study

Gene/transcript^*^	Variant location	Variant	Chromosome position (GRCh37)	Zygosity^1^	OMIM number^2^	Inheritance pattern^3^	Variant Classification^4^
*MITF* ENST00000352241.4 NM_198159.3	Exon 9	c.1026dup p.(Asn343Glufs*27)	Chr3: 70,008,434_70,008,435	Het	193,510	AD	Pathogenic

*Ensemble Transcript ID starts with ENST, RefSeq (NCBI) Transcript ID starts with NM

^1^Het: Heterozygous

^2^OMIM number: Six-digit number assigned to each phenotype in Online Mendelian Inheritance in Man (OMIM) database

^3^AD: Autosomal dominant

^4^Based on American College of Medical Genetics and Genomics (ACMG) standards and guidelines for the interpretation of sequence variants, 2015

This variant is absent in gnomAD [20] and 1000 genome [21] database and was not found in our local database that contains almost 2500 WES data. The variant is not reported in dbSNP [22] database as well and no former record of this variant is available in Clinvar [23], Human Gene Mutation Database (HGMD) [24], and Online Mendelian Inheritance in Man (OMIM) [25] databases. This confirms that a novel variant was reported here for the first time. PCR-Sanger sequencing also affirmed this heterozygous variant in the patient.

**Fig. 3:**
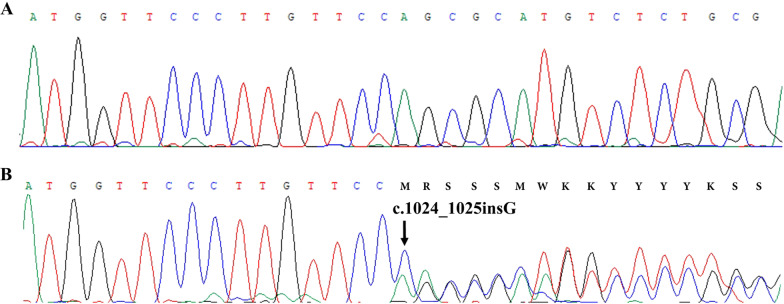
**A** Partial DNA sequence chromatogram of exon 9 of the *MITF* gene (ENST00000352241.4) in a healthy individual II-3. **B** Partial DNA sequence chromatogram of exon 9 of the *MITF* gene with the frameshift mutation c.1026dup p.(Asn343Glufs*27) in heterozygous state in patient I-1. The black arrow shows the c.1026dup mutation. M: A/C, R: G/A, S: G/C, W: A/T, K: G/T, Y: T/C (sequencing is shown by reverse primer)

Segregation analysis at the pedigree level revealed co-segregation of this variant with the disease in all affected individuals examined in the family and was not observed in healthy family members. Thus, the presence of the identified mutation was confirmed in proband (II-2), his two brothers (II-7 and II-9), two sisters (II-11 and II-13), niece (III-8), and mother (I-1) by PCR-Sanger sequencing, but the mutation was not found in unaffected individuals (II-3 and III-7) (Fig. 3). According to these observations and ACMG guidelines for variant interpretation, this variant was classified as a pathogenic variant. Therefore, genetic diagnosis of phenotype in this family is confirmed.

## Discussion

**Fig. 4:**
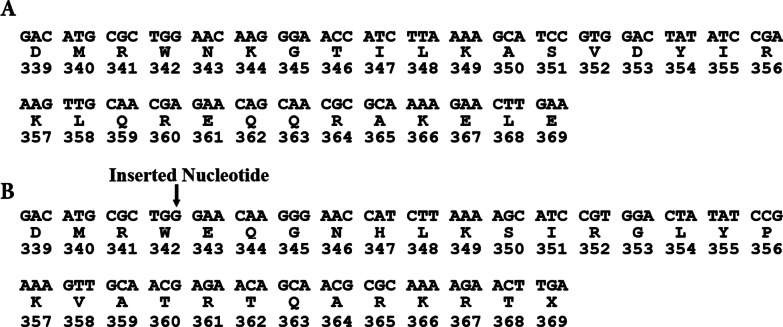
**A** portion of nucleotide and deduced amino acid sequences of exon 9 of the *MITF* gene. **B** c.1026dup in exon 9 of the *MITF* gene caused a frameshift mutation that ultimately resulted in an early termination which is expected

As an auditory-pigmentary syndrome, Waardenburg syndrome occurs with an estimate of 1:40,000 in the general population and accounts for 2–5% of congenital deafness [26, 27]. There are 520 amino acid residues in MITF isoform A, expressed in multiple cell types such as melanocytes and retinal pigment epithelium (RPE) cells. The present study describes clinical features and mutation detection in the *MITF* gene of WS2 patients in a large Iranian family. A novel frameshift heterozygous mutation, c.1026dup p.(Asn343Glufs*27), was found in the proband. The variant had co-segregation with the whole affected members examined in this family and was not observed in healthy individuals. This mutation was a single-base insertion of a G after base 1024 within the loop of the MITF protein, leading to a frameshift and a stop codon, 27 codons downstream from the insertion point. Therefore, it is truncated within the leucine zipper without 23 residues of the leucine zipper portion, and gives rise to a truncated MITF protein with 368 of the 520 wild type amino acids (Fig. 4). Accordingly, eliminated protein length has been circa 30% [11, 28, 29]. No dystopia canthorum, musculoskeletal abnormalities or Hirschsprung disease were detectable in affected individuals of this family and the pedigree chart demonstrates autosomal dominant heredity. According to Waardenburg Consortium, clinical manifestations, and the variant discovered in *MITF* gene in our patients diagnosis of WS2A was affirmed [30]. The bHLHZip structure of MITF has an important role in its activity. Apparently, a significant function of the leucine zipper is in dimerization as its deletion can head to loss of function at the level of dimerization and DNA binding. In the present research, the mutant MITF protein does not have some residues of the leucine zipper portion for its dimerization with wild type MITF protein, thereby impairing its normal function. Consequently, this may influence the expression of its target genes, leading to the lack of normal melanocytes in skin, eye, and cochlea [2, 13]. Previously, it was reported that a heterozygous nonsense mutation, c.859G > T p.(E287X), in exon 9 within the leucine zipper of MITF in affected members of a single family led to WS2 [12].

Mutations of Ser298 in MITF-M (Ser399 in MITF-A), situated downstream of bHLHZip, were found to have a major contribution to transcriptional activity of MITF. In an in vitro investigation, phosphorylation of Ser298 by Glycogen synthase kinase 3 (GSK3) was shown to improve the ability of MITF to bind the DNA. Therefore, phosphorylation of MITF by GSK3β in this truncated protein is abolished that impairs the activity of MITF, which can potentially induce WS2. Moreover, serine-rich and threonine-rich regions of MITF are of importance for its activity. Hence, transactivation assays indicated that the truncation of the last 95 amino acids (comprising threonine- and serine-rich regions) reduced the transcriptional activity of MITF (MITF-M[1–324], MITF-A[1–425]. Transactivation and DNA-binding assays demonstrated that the deletion of (MITF-M[294–324], MITF-A[395–425]), which is downstream of the bHLHZip structure, reduced the transactivation activity and the ability of MITF to bind the E-box respectively. As a result, the last 95 amino acids (comprising threonine- and serine-rich regions) are absent in the truncated MITF protein in our study, which may influence its transcriptional activity. Additionally, deletion of the amino acids from 395 to 425—downstream of the bHLHZip structure—reduced the transactivation and DNA binding ability of the MITF protein to bind the E-box sequence [13, 28] Here, the location of the detected mutation is in the last translated exon, hence, it is predictable to cause the mutant transcript to escape from the nonsense-mediated mRNA decay (NMD) pathway [5, 31]. Nobukuni et al. reported that the MITF proteins with truncated mutations within bHLHZip region were not capable of dimerization with wild-type MITF proteins, but they showed no interference with DNA binding ability of wild-type MITF protein. Thus, loss of function mutations cause the clinical characteristics of patients with WS2A, which result in haploinsufficieny of the MITF protein [2]. Haploinsufficiency is apparently a key mechanism in deafness and pigmentary abnormalities of WS2 patients [13].

Tietz syndrome (TS) (OMIM 103500) is a more severe form of WS2A, the characteristics of which are profound congenital deafness and generalized hypopigmentation of skin, hair, and eye that is transferred in an autosomal dominant manner with heterozygous mutations within the *MITF* gene. WS type 2A, on the other hand, is discriminated from TS by patchy depigmentation abnormalities of skin, hair, and irides [8, 9]. A less severe phenotype with no generalized hypopigmentation of skin, hair, and eye was observed in our patients with *MITF* mutation. Expressivity varies in patients with WS2A [8]. Thus, variable phenotypes were noticed amongst patients of this family. Typically, WS2 is characterized by sensorineural hearing loss and heterochromia iridum [7]. Clinically, hearing loss (7/7, 100.0%) was the most common feature detected amongst affected individuals of this family, but it varied from mild to profound. In the current research, pigmentary disturbances of iris were highly frequent, which affected seven out of the seven patients, namely complete heterochromia iridis in one patient (II-2), partial or segmental heterochromia of one or both eyes in three patients (II-7, II-9 and II-11), and brilliant blue irides in three patients (I-1, II-13 and III-8). Freckles on skin are a phenotypic feature with more frequency in skin pigmentary disorders in Asian population than in western patients [7, 32]. As a result, affected individuals of this family presented a striking frequency of numerous brown freckles (6/7, 85.71%). Synophrys occurred in (5/7, 71.42%) of the studied patients and early graying prior to age 30 (4/7, 57.14%) had the lowest frequency amongst our patients.

According to the American College of Medical Genetics and Genomics (ACMG) standards and guidelines 2015 [33], the variant c.1026dup p.(Asn343Glufs*27) of *MITF* was considered to be pathogenic. Table 2 represents the impact of this variant on all transcripts of the *MITF* gene (data derived from Varsome genome interpretation tool, varsome.com) [34]. Exploration of the precise mechanisms for the pathogenesis of this mutation requires functional analysis. The detected heterozygous frameshift insertion variant in *MITF* gene is not present in previous reports for its pathogenicity and it is absent in population databases, including Exome Aggregation Consortium (ExAC) (http://exac.broadinstitute.org/), 1000 Genomes (http://www.1000genomes.org/), dbSNP (http://www.ncbi.nlm.nih.gov/snp/) and our local database.

**Table 2:** Pathogenic mutation c.1026dup p.(Asn343Glufs*27) with regard to all isoforms

Transcript ID	Coding impact	Gene	HGVS coding	HGVS Protein	Location	Protein length
ENST00000352241.4	Frameshift	MITF	c.1026dup	N343Efs*27 p.(Asn343Glufs*27)	Exon 9	520aa
ENST00000314557.6	Frameshift	MITF	c.705dup	N236Efs*27 p.(Asn236Glufs*27)	Exon 8	413aa
ENST00000314589.5	Frameshift	MITF	c.978dup	N327Efs*27 p.(Asn327Glufs*27)	Exon 9	504aa
ENST00000328528.6	Frameshift	MITF	c.1023dup	N342Efs*27 p.(Asn342Glufs*27)	Exon 9	519aa
ENST00000394351.3	Frameshift	MITF	c.723dup	N242Efs*27 p.(Asn242Glufs*27)	Exon 8	419aa
ENST00000394355.2	Frameshift	MITF	c.951dup	N318Efs*27 p.(Asn318Glufs*27)	Exon 8	495aa
ENST00000448226.2	Frameshift	MITF	c.1044dup	N349Efs*27 p.(Asn349Glufs*27)	Exon 9	526aa
ENST00000451708.1	Frameshift	MITF	c.996dup	N333Efs*27 p.(Asn333Glufs*27)	Exon 9	361aa
ENST00000472437.1	Frameshift	MITF	c.870dup	N291Efs*27 p.(Asn291Glufs*27)	Exon 9	468aa
ENST00000531774.1	Frameshift	MITF	c.537dup	N180Efs*27 p.(Asn180Glufs*27)	Exon 8	357aa

## Conclusions

In the present study, a novel variant, c.1026dup p.(Asn343Glufs*27), in exon 9 of *MITF* gene was identified in a young male patient and his affected family members. This frameshift mutation ultimately leads to an early termination, giving rise to the generation of a truncated MITF protein that is able to influence the typical activity of the protein. Exactly described mutations involved in WS offer helpful information to elucidate the molecular cause of clinical features of WS and have a contribution to better genetic counseling of WS patients and their families.

## Supplementary Information

**Additional file 1.** Peer review reports.

## Data Availability

The datasets generated and/or analysed during the current study are available in the ClinVar (accession number: SCV001481852; https://www.ncbi.nlm.nih.gov/clinvar/variation/997716/) and Leiden Open Variation Database (LOVD; https://databases.lovd.nl/shared/individuals/00333431).

## Abbreviations

WS: Waardenburg syndrome
MITF: Melanocyte inducing transcription factor
SOX10: SRY-box transcription factor 10
SNAI2: Snail family transcriptional repressor 2
EDNRB: Endothelin receptor type B
EDN3: Endothelin 3
PAX3: Paired box 3
MiT: Microphthalmia
bHLHZip: Basic-helix-loop-helix-leucine zipper
TAD: Transactivation domain
TYR: Tyrosinase
TRP1: Tyrosinase-related protein 1
WES: Whole exome sequencing
GSK3: Glycogen synthase kinase 3
NMD: Nonsense-mediated mRNA decay
TS: Tietz syndrome
