# Supplementary material for: Waardenburg syndrome type 2A in a large Iranian family with a novel MITF gene mutation
Source: BMC Med Genomics. 2021 Sep 20;14:230. doi: 10.1186/s12920-021-01074-y (PMC8451132; doi:10.1186/s12920-021-01074-y)
Supplement: Supplementary file 1 — Additional file 1. Peer review reports. [file 12920_2021_1074_MOESM1_ESM.zip › MGNM-D-20-00433 - Round 1 Ref reports & AU responses.docx]

MGNM-D-20-00433 (Original Submission)

04f311c6-d02a-45a9-8d22-9cf78d188beb (final submission)

**Review round 1**

Reviewer 1:

Dear authors,

in general I have no remarks that would speak against the publication of your article. However, I have several points that should be improved.

Please find enclosed my comments and proposed changes:

According to HGVS, the correct notation is for you mutation should be NM_198159.3:c.1026dup

Please use the bracktes for the potential protein effect throughout the manuscript --> p.(Asn343Glufs*27)

exon 9 common to all isoforms, while the cDNA, amino acid position is not. And it's not "exon 9" for all transcripts. Please clarify this.

Also here: "MITF mutations are located in exons 7, 8 and 9 that encode the bHLH-zip domain [3, 25]."

>silico computational analysis (Mutation Taster, CADD, etc.)

Please remove "etc." from the manuscript and mention all used tools or rephrase the sentence accordingly.

>Our results show that a nucleotide insertion, c.1024_1025insG, in exon 9 of MITF gene causes a frameshift that ultimately results in an early termination

You have no experimental evidence that this leads to early termination. Although it is quite likely. Please phrase more carefully.

Methods:

>Nearly all exons and flanking 10bp were detected and analyzed. Detected variations include single point mutations, and small indels (within 20bp).

What does "nearly all" means? Please give numbers. Which software and paramters were used to align the reads? How are the variants called? Software? Paired-end Sequencing? Read length?

>Multiple lines of in silico computational analysis such as MutationTaster (http://www.mutationtaster.org), CADD (http://cadd.gs.washington.edu), etc.

Please give all used or relevant databases, software with version/release. Please cite the corresponding publications. Why are you using ExAC but not gnomAD?

>1000 Genomes (http://www.1000genomes.org/), dbSNP (http://www.ncbi.nlm.nih.gov/snp /) and our local database.

How many samples are in your database? Without further details it makes no sense to mention it.

Please show or discuss the pathogen mutation with regard to all isoforms. For MITF several isoforms have been described (MITF‐A, MITF‐B, MITF‐C, MITF‐D, MITF‐E, MITF‐H, MITF‐J, MITF‐M) with differential expression patterns in humans (Kawakami & Fisher, 2017).

Which isoform are you talking about?

>Figure 3. The DNA sequence chromatogram of exon 9 of the MITF gene

In the figure is not the complete sequence of exon 9. Please add also the transcriptID to which you refer.

Please revise carefully the text. There are some sentences that could be improved e.g. The mutation found in MITF gene seems that disrupted the formation of the dimers necessary for its proper function and impaired the function of MITF protein.

Reviewer 2: In this study, Zardadi et al identified a novel frameshift MITF mutation in a Waardenburg syndrome type 2A pedigree. The data is clear and convincing, however, I have following concerns.

1. "Table 1: Identified variant in in this study", the "in" is duplicated.

2. In Figure 1, please indicate the meaning for I, II, III; 1, 2, 3; and symbols.

3. In Figure 1, there are numbers in the individuals III:1, III:2, and III:7, please explain the meanings for these numbers in the figure legend.

4. In Figure 3, the authors should provide the chromatogram from an unaffected individual.

5. In Figure 3, please provide the meaning for the letters M, R, S, W, K, Y on the top.

6. Figure 3 is not clear enough to understand the insertion and its consequences, please refer to the figure 2 in Mosher et al (2007) PLoS Genet 3(5): e79. doi:10.1371/journal.pgen.0030079.

**Author responses to round 1**

Journal of BMC Medical Genomics

Dear Editor-in-chief,

It is with excitement that we resubmit a revised version of the manuscript “Waardenburg syndrome type 2A in a large Iranian family with a novel MITF gene mutation”. Thank you for giving us the opportunity. We carefully considered your suggestions as well as those offered by the reviewers.

We have responded specifically to each suggestion below and to make the changes easier to identify in the main manuscript, all changes are highlighted in yellow. We appreciate the time and detail provided by you and each reviewer and has incorporated the suggested changes into the manuscript to the best of our ability. The manuscript has certainly benefited from these insightful revision suggestions.

The revision has been developed in consultation with all co-authors, and each author has approved the final form of this revision. Furthermore, in agreement with all authors, we added the name of Professor Mohammad Keramatipour, Department of Medical Genetics, Faculty of Medicine, Tehran University of Medical Sciences, Tehran, Iran to the present manuscript for his cooperation in revision of the manuscript. The signed authorship form was also attached.

We very much hope the revised manuscript will be accepted for publication in your journal.

Sincerely yours,

Saeid Morovvati, PhD, Department of Genetics, Faculty of Advanced Sciences and Technology, Tehran Medical Sciences, Islamic Azad University, Tehran, Iran.

Tel: +98-21-71365

Fax: +98-21-77445377

Email: morovvati@iautmu.ac.ir, morovvati@hotmail.com

Reviewer 1

According to HGVS, the correct notation is for you mutation should be NM_198159.3:c.1026dup

Thank you for your comments. We used the correct notation for the mutation in these sections:

Abstract section, line 31, page 2

Abstract section, line 35, page 2

Discussion section, line 197, page 10

Discussion section, line 258, page 12

Conclusions section, line 267, page 13

Figure legend section, figure 3

Table 1

Please use the brackets for the potential protein effect throughout the manuscript

p.(Asn343Glufs*27)

We used brackets for the potential protein effect throughout the manuscript in these sections:

Abstract section, line 31, page 2

Discussion section, line 197, page 10

Discussion section, line 215, page 11

Discussion section, line 258, page 12

Conclusions section, line 267, page 13

Figure legend section, figure 3

Table 1

exon 9 common to all isoforms, while the cDNA, amino acid position is not. And it’s not ̎ exon 9 ̎ for all transcripts. Please clarify this. Also here: ̎ MITF mutations are located in exons 7, 8 and 9 that encode the bHLH-zip domain [3, 25]. ̎

The mutation (NM_198159.3:c.1026dup) identified in this study was in MITF-A gene so we deleted the sentence ̎ MITF mutations are located in exons 7, 8 and 9 that encode the bHLH-zip domain [3, 25] ̎ from our manuscript because this sentence was not in relation to MITF-A, so reference 25 was deleted.

silico computational analysis ( Mutation Taster, CADD, etc.)

Please remove "etc." from the manuscript and mention all used tools or rephrase the sentence accordingly.

The manuscript was corrected in method section, line 109-137, page 6-7 and results section, line 152-183, page 8-9

Our results show that a nucleotide insertion, c.1024_1025insG, in exon 9 of MITF gene causes a frameshift that ultimately results in an early termination. You have no experimental evidence that this leads to early termination. Although it is quite likely. Please phrase more carefully.

We fully agree with the reviewer so in figure 4 we showed that the frameshift mutation c.1026dup leads to early termination and the sentences ̎ Figure 4. A) portion of nucleotide and deduced amino acid sequences of exon 9 of the MITF-A gene. B) c.1026dup in exon 9 of the MITF-A gene caused a frameshift mutation that ultimately resulted in an early termination which is expected ̎ were added in Figure legend section, figure 4

Methods:

Nearly all exons and flanking 10bp were detected and analyzed. Detected variations include single point mutations, and small indels (within 20bp).

What does "nearly all" means? Please give numbers. Which software and paramters were used to align the reads? How are the variants called? Software? Paired-end Sequencing? Read length?

Multiple lines of in silico computational analysis such as MutationTaster (http://www.mutationtaster.org), CADD (http://cadd.gs.washington.edu), etc.

Please give all used or relevant databases, software with version/release. Please cite the corresponding publications. Why are you using ExAC but not gnomAD?

1000 Genomes (http://www.1000genomes.org/), dbSNP (http://www.ncbi.nlm.nih.gov/snp /) and our local database.

How many samples are in your database? Without further details it makes no sense to mention it.

The manuscript was corrected in method section, line 109-137, page 6-7 and results section, line 152-183, page 8-9

Please show or discuss the pathogen mutation with regard to all isoforms. For MITF

several isoforms have been described (MITF-A, MITF-B, MITF-C, MITF-D, MITF-

E, MITF-H, MITF-J, MITF-M) with differential expression patterns in humans

(Kawakami & Fisher, 2017).

Which isoform are you talking about?

According to varsome (varsome.com) database we showed the pathogen mutation with regard to all isoforms in Table 2 and the sentence ̎ According to varsome (varsome.com) database this pathogenic mutation with regard to all isoforms has been shown in Table 2.̎ was added in discussion section, line 259-260, page 12-13.

According to varsome (varsome.com), ncbi (http://www.ncbi.nlm.nih.gov) and ensemble (www.ensemble.org) databases and also, with referring to the published article of Amae et al in Biochem Biophys Res Commun. 1998 Jun 29;247(3):710-5. doi: 10.1006/bbrc.1998.8838. PMID: 9647758, the pathogenic mutation of this study [NM_198159.3:c.1026dup p.(Asn343Glufs*27) , ENST00000352241.4 ] occurs in MITF isoform A. Also we added the sentence ̎ MITF isoform A consists of 520 amino acid residues expressed in many cell types including melanocytes and retinal pigment epithelium (RPE) cells ̎ in discussion section, line 194-195, page 10, so the reference 23 was added.

Figure 3. The DNA sequence chromatogram of exon 9 of the MITF gene

In the figure is not the complete sequence of exon 9. Please add also the transcriptID to

which you refer

̎ The DNA sequence chromatogram of exon 9 of the MITF gene with the frameshift mutation c.1024_1025insG (p.Asn343Glufs*27) in heterozygous state in patient I-1. The black arrow above the chromatogram sequence shows the c. 1024_1025insG mutation ̎ in figure legend section, figure 3 was corrected to ̎ A) Partial DNA sequence chromatogram of exon 9 of the MITF-A gene (ENST00000352241.4) in a healthy individual II-3. B) Partial DNA sequence chromatogram of exon 9 of the MITF-A gene with the frameshift mutation c.1026dup p.(Asn343Glufs*27) in heterozygous state in patient I-1. The black arrow shows the c.1026dup mutation. M: AC, R: GA, S: GC, W: AT, K: GT, Y: TC (sequencing is shown by reverse primer). ̎

Please revise carefully the text. There are some sentences that could be improved e.g. The mutation found in MITF gene seems that disrupted the formation of the dimers necessary for its proper function and impaired the function of MITF protein.

Thank you for your comments. We revised the text carefully.

1- MITF in [Abstract section, line 23, 32, 35, page 2; background section, line 73, page 4; methods section, line 139, page 7; discussion section, line 196 and 207, page 10 and line 258, page 12 and 262, page 13, conclusions section, line 267, page 13, figure legend section, figure 1 and table 1] was corrected to MITF-A.

2- ̎Multiple lines of in silico computational analysis (Mutation Taster and CADD) support deleterious effect of this variant on the gene or gene product(s) ̎ in abstract section, line 32-34, page 2 was corrected to ̎ Mutation Taster predicted this variant as a disease causing variant. UniProt has reported this variant in mutational hot spot and critical and well-established functional domain of related protein ̎.

3- ̎ and ̎ in background section, line 60, page 3 was deleted.

4- Multiple lines of in silico computational analysis such as MutationTaster (http://www.mutationtaster.org), CADD (http://cadd.gs.washington.edu), etc. were used to predict the pathogenicity of the variant was deleted in method section, line 149, page 7.

5- ̎ Thus, it is truncated within the leucine zipper lacking 21 residues of the leucine zipper portion ̎ in discussion section, line 202, page 10 was corrected to ̎ Thus, it is truncated within the leucine zipper lacking 23 residues of the leucine zipper portion ̎.

7- ̎ with 369 of the 520 wild type amino acids ̎ in discussion section, line 203, page 10 was corrected to ̎ with 368 of the 520 wild type amino acids ̎.

8- (Fig. 4) was added in discussion section, line 203, page 10.

9- Multiple lines of in silico computational analysis (CADD, Mutation Taster, etc.) support the deleterious effect of the variant on the gene or gene product(s) was deleted in discussion section, line 208, page 10.

10- ̎ The mutation found in MITF gene seems that disrupted the formation of the dimers necessary for its proper function and impaired the function of MITF protein ̎ in discussion section, line 210-212, page 10 was corrected to ̎ in this study, the mutant MITF protein lack some residues of the leucine zipper portion for its dimerization with wild type MITF protein therefore its normal function has been impaired ̎.

11- MITF: microphthalmia-associated transcription factor; SOX10: SRY (sex-determining region Y)-box10; SNAI2: snail homolog of 2; EDN3: endothelin-3; PAX3: paired box gene 3 in Abbreviation section was corrected to MITF: melanocyte inducing transcription factor; SOX10: SRY-box transcription factor 10; SNAI2: snail family transcriptional repressor 2; EDN3: endothelin 3; PAX3: paired box 3 respectively.

Reviewer 2

1- ̎ Table 1: Identified variant in in this study ̎, the ̎ in ̎ is duplicated.

Thank you for your comments. The second ̎ in ̎ was deleted in Table 1.

2- In Figure 1, please indicate the meaning for I, II, III; 1, 2, 3; and symbols.

Each generation is identified by a Roman numeral (I, II, III) and each individual within the same generation is identified by an Arabic numeral (1, 2, 3 and so on). Squares represent males and circles represent females. The symbol with a diagonal line shows a deceased individual. Individuals’ symbols are colored black to indicate the presence of clinical characteristics of WS2, unfilled symbols represent unaffected family members which has been written in Figure legend section, figure 1 with referring to the figure 1 in Polan et al in European Journal of Human Genetics (2014)22,105-109;doi:10.1038/ejhg.2013.99 and figure 1 in Choi et al in BMC Ophthalmology (2018) 18:266.https://doi.org/10.1186/s12886-018-0933-9.

3- In Figure 1, there are numbers in the individuals III:1, III:2, and III:7, please explain the meanings for these numbers in the figure legend.

The numbers inside symbols represent number of individuals which has been written in Figure legend section, figure 1, with referring to the figure 1 in Polan et al article in European Journal of Human Genetics (2014)22,105-109;doi:10.1038/ejhg.2013.99.

4- In Figure 3, the authors should provide the chromatogram from an unaffected individual.

The chromatogram from an unaffected individual was provided in Figure 3.

5- In Figure 3, please provide the meaning for the letters M, R, S, W, K, Y on the top.

The meaning for the letters M, R, S, W, K, Y in figure 3 was provided which has been written in Figure legend section, figure 3.

6- Figure 3 is not clear enough to understand the insertion and its consequences, please refer to the figure 2 in Mosher et al (2007) PloS Genet 3(5): e79. doi: 10.1371/journal .pgen.0030079.

Thank you for your comments. Figure 3 was corrected. And also, figure 4 was provided to show that the frameshift mutation c.1026dup leads to early termination.
