# Supplementary material for: Waardenburg syndrome type 2A in a large Iranian family with a novel MITF gene mutation
Source: BMC Med Genomics. 2021 Sep 20;14:230. doi: 10.1186/s12920-021-01074-y (PMC8451132; doi:10.1186/s12920-021-01074-y)
Supplement: Supplementary file 1 — Additional file 1. Peer review reports. [file 12920_2021_1074_MOESM1_ESM.zip › MGNM-D-20-00433 - Round 2 Ref reports & AU responses.docx]

MGNM-D-20-00433 (Original Submission)

04f311c6-d02a-45a9-8d22-9cf78d188beb (final submission)

**Review round 2**

Reviewer reports:

Reviewer 1: The reviewers' comments were well implemented and improved the manuscript.

The following points should be improved:

1. MITF-A is not a gene. MITF-A is an alternative isoform of the gene MITF. Please correct this throughout the manuscript.

2. Please discuss the "truncated MITF protein" more carefully. Especially in the abstract the impression is given that the manuscript would provide experimental data for a truncated protein.

3. Some information given in the methods/results are redundant an could be removed (i.e. Exome sequencing, variant analysis).

4. "UniProt has reported this variant in mutational hot spot" - Please describe in more detail. This is based on which data in UniProt?

5. Please cite all software, databases used. e.g. Varsome. Please provide version/release information.

Reviewer 2: The authors fully addressed my concerns. I only ask the authors to made the following two small changes :

1. The legend for Figure 2 should be: M: A/C, R: G/A, S: G/C, W: A/T, K: G/T, Y: T/C

2. The format of the table should be three-line format

**Author responses to round 2**

Journal of BMC Medical Genomics

Dear Editor-in-chief,

It is with excitement that we resubmit a revised version of the manuscript “Waardenburg syndrome type 2A in a large Iranian family with a novel MITF gene mutation”. Thank you for giving us the opportunity. We carefully considered your suggestions as well as those offered by the reviewers.

We have responded specifically to each suggestion below and to make the changes easier to identify in the main manuscript, all changes are highlighted in yellow. We appreciate the time and detail provided by you and the reviewers and has incorporated the suggested changes into the manuscript to the best of our ability. The manuscript has certainly benefited from these insightful revision suggestions.

The revision has been developed in consultation with all co-authors, and each author has approved the final form of this revision. We very much hope the revised manuscript will be accepted for publication in your journal.

Sincerely yours,

Saeid Morovvati, PhD, Department of Genetics, Faculty of advanced sciences and Technology, Tehran Medical Sciences, Islamic Azad University, Tehran, Iran.

Reviewer 1

1. MITF-A is not a gene. MITF-A is an alternative isoform of the gene MITF. Please correct this throughout the manuscript.

Thank you for your comments. MITF-A was corrected to MITF throughout the manuscript.

2. Please discuss the ̎ truncated MITF protein ̎ more carefully. Especially in the abstract the impression is given that the manuscript would provide experimental data for a truncated protein.

̎ Our results show that a nucleotide insertion c.1026dup, in exon 9 of MITF-A gene causes a frameshift that ultimately results in an early termination, leading to formation of a truncated protein that can affect the normal function of the MITF protein. ̎ in abstract section, line 36-38, page 2, was corrected to ̎ A nucleotide insertion c.1026dup, in exon 9 of the MITF gene causes a frameshift. This mutation can cause an early termination, leading to formation of a truncated protein that can affect the normal function of the MITF protein. ̎

̎ As a result, the truncated MITF protein in this study does not have the last 95 amino acids and also the amino acids from 395 to 425 that can affect its transcriptional activity, transactivation capacity and DNA binding ability ̎ in discussion section, line 192-196, page 9-10, was corrected to ̎ As a result, the truncated MITF protein in this study does not have the last 95 amino acids (including threonine- and serine-rich regions) that can affect its transcriptional activity. Moreover, deletion of the amino acids from 395 to 425 -downstream of the bHLHZip structure- decreased the transactivation capacity and DNA binding ability of MITF protein to bind the E-box sequence. ̎

3. Some information given in the methods/results are redundant and could be removed (i.e. Exome sequencing, variant analysis).

Most of previous text was removed and only essential information stored.

4. ̎ Uniprot has reported this variant in mutational hot spot ̎ - please describe in more detail. This is based on which data in Uniprot?

The location of variant on protein functional domain, based on Uniprot database, was added into the article in abstract section, line 32-35, page 2 and results section, line 137-140, page 7.

5. Please cite all software, databases used. e.g. Varsome. Please provide version/ release information.

Thank you for your comments. Databases and softwares were cited as recommended by their providers.

Reviewer 2

1. The legend for Figure 2 should be: M: A/C, R: G/A, S: G/C, W: A/T, K: G/T, Y: T/C

Thank you for your comments. The legend for Figure 2 was corrected

2. The format of the table should be three-line format.

The format of the tables was corrected.
